# LncRNA RP11-89 facilitates tumorigenesis and ferroptosis resistance through PROM2-activated iron export by sponging miR-129-5p in bladder cancer

**DOI:** 10.1038/s41419-021-04296-1

**Published:** 2021-11-02

**Authors:** Wenjie Luo, Jun Wang, Wenhao Xu, Chunguang Ma, Fangning Wan, Yongqiang Huang, Mengfei Yao, Hailiang Zhang, Yuanyuan Qu, Dingwei Ye, Yiping Zhu

**Affiliations:** 1 Department of Urology, Fudan University Shanghai Cancer Center, Shanghai, China; 2 Department of Oncology, Shanghai Medical College, Fudan University, Shanghai, China; 3 Department of Urology, Sun Yat-sen University Cancer Center, Guangzhou, China

**Keywords:** Tumour biomarkers, Tumour biomarkers

## Abstract

Long non-coding RNAs (lncRNAs) act as important regulators of tumorigenesis and development in bladder cancer. However, the underlying molecular mechanisms remain elusive. We previously identified a novel lncRNA signature related to immunity and progression in bladder cancer. Here we further explored the function of RP11-89, a lncRNA discovered in the previous signature. Loss- and gain-of function experiments were performed using CCK-8 assay, flow cytometry, Transwell assays, scratch tests and subcutaneous nude mouse models. High-throughput RNA sequencing was conducted to identify dysregulated genes in bladder cancer cells with RP11-89 knockdown or overexpression. Regulation of RP11-89 on miR-129-5p and PROM2 was explored through luciferase reporter assay, RIP assay and RNA pull-down assay. RP11-89 promoted cell proliferation, migration and tumorigenesis and inhibited cell cycle arrest via the miR-129-5p/PROM2 axis. We found that RP11-89 “sponges” miR-129-5p and upregulates PROM2. Elevated PROM2 in cells was associated with attenuated ferroptosis through iron export, formation of multivesicular bodies and less mitochondrial abnormalities. We demonstrated that RP11-89 is a novel tumorigenic regulator that inhibits ferroptosis via PROM2-activated iron export. RP11-89 may serve as a potential biomarker for targeted therapy in bladder cancer.

## Introduction

As one of the ten most frequent malignancies in the world, bladder cancer (BLCA) is the most lethal urogenital tumor, with approximately 573,000 new cases and 213,000 deaths in 2020 [1]. Accumulating evidence has shown that microRNAs (miRNAs) and long noncoding RNAs (lncRNAs) play key roles in the genesis, progression, and treatment of BLCA [2, 3]. Better understanding of the molecular mechanisms underlying BLCA is critical for the development of new treatments.

LncRNAs are autonomously transcribed non-coding RNAs longer than 200 nucleotides that do not overlap annotated coding genes [4]. An increasing number of lncRNAs have been investigated over the past decade for their roles in multiple cancers [5]. For instance, the lncRNA RBAT1 is highly expressed in both retinoblastoma and BLCA and associated with tumorigenesis in vitro and in vivo [6]. Furthermore, lncRNAs exert regulatory interactions on miRNAs through acting as competing endogenous RNAs (ceRNAs). These efficient miRNA sponges contribute to epigenetic modifications and alter cancer malignant phenotypes such as proliferation and therapeutic resistance.

Ferroptosis, a form of cell death that differs from apoptosis [7], has attracted increasing attention in recent years. Ferroptosis is currently acknowledged as one of the most widespread and ancient forms of cell death [8, 9]. The accumulation of iron, fatty acid supply, and lipid peroxidation induces cellular ferroptosis, resulting in iron-mediated oxidative damage of cell membranes such as the inner mitochondrial membrane with an increasing level of reactive oxygen species (ROS) [10–12]. This kind of ferroptotic injury plays an important role in the fate of tumor cells via damage-related molecular patterns and multiple cancer pathways [13, 14]. For example, Zhang et al. reported that ferroptosis regulates the tumor microenvironment by decreasing TAF activation and reducing TGF-β secretion in breast cancer [13]. Evidence has shown that crucial regulatory proteins in ferroptosis such as GPX4 [15, 16] and SLC7A11 [17, 18] also function as important triggers in the regulation of tumor-related signaling mechanisms such as tumorigenesis of bladder cancer. For example, Liu demonstrated that SLC7A11 could be regulated by OTUB1 in cancer cells and facilitate ferroptosis resistance in bladder cancer using T24 Cell and UM-UC 3 Cell as experimental models [19].

In addition, clinical application of immune checkpoint inhibitors could be greatly expanded when combined with ferroptosis strategies such as anti-PD-L1 agents [20, 21]. The immunogenic cell death that contributes to antitumor immune surveillance may be regulated by ferroptosis [22]. For example, CD36 reduced cytotoxic cytokine production and impaired antitumor ability of tumor-infiltrating CD8 T cells via inducing ferroptosis [23]. As we know, the heterogeneity of immune environment in BLCA is particularly high. Patients with high CD8 T cell infiltration are associated with better outcomes in response to immune checkpoint blockade therapy such as anti-PD1 therapy [24]. Interestingly, Wang et al. have pointed out that immunotherapy-activated CD8 T cells contribute to the anti-tumor efficacy via enhancing ferroptosis in tumor cells [20]. There exists a tight association between ferroptosis and tumor immune response.

We previously identified a novel prognostic lncRNA signature related to the immune environment for BLCA by systematic bioinformatic discovery, and RP11-89, one of lncRNAs in the signature, was found to play an oncogenic role in BLCA through inhibiting regulated cell death [25]. In the current study, we focused on the regulation of ferroptosis resistance by RP11-89 and further investigated the role of RP11-89 in the tumorigenesis of BLCA. RP11-89 has the potential to be a novel target for BLCA ferroptosis strategy.

## Results

### RP11-89 expression is upregulated in BLCA and associated with ferroptosis

**Fig. 1:**
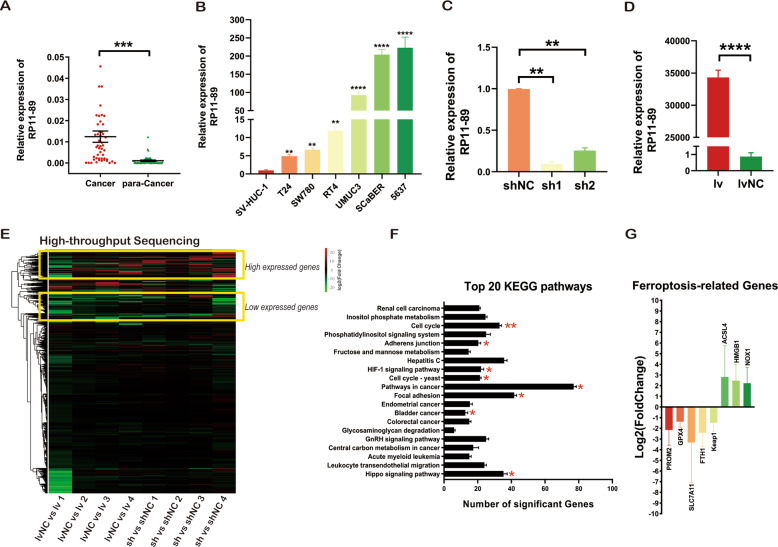
Dysregulated expression of RP11-89 and its association with ferroptosis in BLCA tumorigenesis. **A** RP11-89 expression in 49 paired BLCA and normal bladder tissues. **B** RP11-89 expression in BLCA cell lines and the SV-HUC 1 cell line (an immortalized human normal urothelial cell line). **C** RT-qPCR showed the knockdown efficiency of sh1-RP11-89 and sh2-RP11-89 shRNA plasmids in the 5637 Cell line. **D** RT-qPCR showed the overexpression efficiency of lv-RP11-89 and lvNC-RP11-89 plasmids in the T24 Cell line. **E** Heatmap depicting the log2FC values of differentially expressed genes from high-throughput sequencing of sh1-RP11-89 5637 Cell and shNC-RP11-89 5637 Cell as well as lvNC-RP11-89 T24 Cell and lv-RP11-89 T24 Cell in four pairs of samples. **F** Top 20 KEGG analysis suggested that RP11-89 exhibits roles in BLCA tumorigenesis via multiple pathways. **G** Histogram showing log2FC values of ferroptosis-related genes including downregulated and upregulated genes. Results are presented as mean ± SD. *P < 0.05; **P < 0.01, ***P < 0.001, ****P < 0.0001. Data were obtained from at least three independent experiments.

Analysis of the public dataset (TCGA-BLCA cohort and GSE89006 in GEO database) suggested that RP11-89 was upregulated in BLCA and closely associated with prognosis of BLCA patients in the previous study [25]. As shown in Fig. 1A, B, compared with para-cancer tissues, BLCA tissues showed markedly high expression of RP11-89, and compared with SV-HUC-1 cells, BLCA cell lines exhibited up-regulated RP11-89 levels. Further analysis of the correlation between the clinicopathological characteristics of BLCA patients from FUSCC cohort and the expression level of RP11-89 expression showed that RP11-89 expression was significantly associated with patient age (*P* = 0.0152), tumor grade (*P* = 0.0134), and lymph node metastasis (*P* = 0.0203) (Table 1).

**Table 1:** Correlation between clinicopathological characteristics of 49 BLCA patients and RP11-89 expression level in FUSCC cohort.

Characteristics	LncRNA RP11-89 expression		*P*-value
	High expression (*N* = 25)	Low expression (*N* = 24)	
*N* (%)			
Age			0.0152*
<70 years	18 (72.0)	9 (37.5)	
≥70 years	7 (28.0)	15 (62.5)	
Gender			0.0723
Male	24 (96.0)	19 (76.1)	
Female	1 (4.0)	5 (23.9)	
Tumor stage			0.2987
I–II	19 (76.0)	21 (79.2)	
III–IV	6 (24.0)	3 (20.8)	
T stage†			0.7263
T1 – T2	21 (84.0)	21 (83.3)	
T3 – T4	4 (16.0)	3 (16.7)	
N stage†			0.091
N0-1	20 (80.0)	23 (95.9)	
N2-3	5 (20.0)	1 (4.1)	
M stage^†^			0.3222
M0	24 (96.0)	24 (100,0)	
M1	1 (4.0)	0 (0)	
Grade			0.0134*
Low	2 (8.0)	9 (37.5)	
High	23 (92.0)	15 (62.5)	
Lymph metastasis			0.0203*
Yes	9 (36.0)	2 (8.3)	
No	16 (64.0)	22 (91.7)	
Subtype			0.3222
Papillary	24 (96.0)	24 (100.0)	
Non-papillary	1 (4.0)	0 (0.0)	

^†^TNM scoring system: tumor size, lymph nodes affected, metastases. *P-value < 0.05.

The efficiency of knockdown and overexpression of RP11-89 with lentivirus infection was confirmed by RT-qPCR (Fig. 1C, D). We performed RNA-sequencing to examine differentially expressed genes in the sh-RP11-89 group and shNC-RP11-89 group as well as the lv-RP11-89 group and lvNC-RP11-89 group (Fig. 1E). As shown in Fig. 1F, KEGG analysis indicated that RP11-89 is associated with pathways in cancer including the cell cycle, adherens junction, HIF-1 signaling pathway, focal adhesion, and Hippo signaling pathway. The fold change in expression of ferroptosis related genes between indicated groups is shown as log2FC (Supplementary Table S1). As shown in Fig. 1G, PROM2, GPX4, SLC7A11, FTH1, and Keap1, which are key ferroptosis-suppressor proteins, showed lower gene expression in sh-RP11-89 cells compared with controls and higher gene expression in lv-RP11-89 cells compared with controls. In addition, ACSL4, HMGB1, and NOX1, which are key ferroptosis-driver proteins, showed higher gene expression in sh-RP11-89 cells compared with controls and lower expression in lv-RP11-89 cells compared with controls. The results revealed that RP11-89 might affect tumor ferroptosis in the regulation of BLCA progression.

### RP11-89 promotes BLCA tumorigenesis in vitro and vivo

**Fig. 2:**
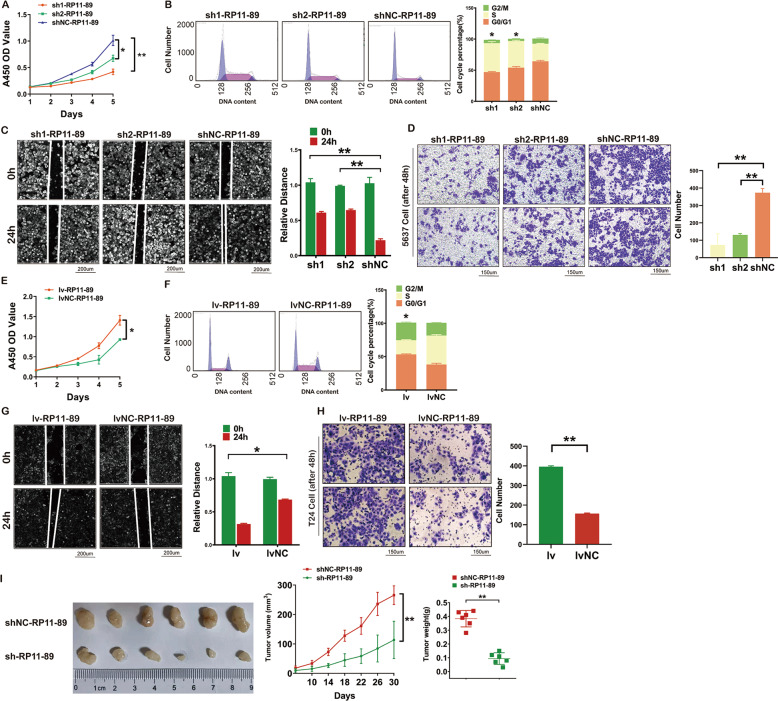
RP11-89 functions as an oncogene in BLCA. **A**, **E** Cell proliferation of BLCA cells with RP11-89 knockdown (**A**) and overexpression (**E**) was assessed by CCK-8 assay. **B**, **F** Cell cycle analysis showed the percentage of G2/M phase, S phase, and G0/G1 phase cells of BLCA cell lines with RP11-89 knockdown (**B**) and overexpression (**F**). **C**, **G** Scratch assays evaluated the migration ability of 5637 Cell with RP11-89 knockdown (**C**) and T24 Cell with RP11-89 overexpression (**G**). **D**, **H** Transwell assays confirmed that migration ability of BLCA cell line was suppressed with RP11-89 knockdown (**D**) and enhanced with RP11-89 overexpression (**H**). **I** Representative images of tumors from nude mice inoculated with BLCA cells and RP11-89 knockdown decreased tumor volume and tumor weight of nude mice after inoculation. Results are presented as mean ± SD. *P < 0.05; **P < 0.01, ***P < 0.001, ****P < 0.0001. Data were obtained from at least three independent experiments.

We next investigated oncogenic functions of RP11-89 in BLCA. CCK8 assay showed that 5637 Cell infected with sh-RP11-89 lentivirus exhibited attenuated cell viability compared with 5637 Cell infected with shNC-RP11-89 lentivirus (Fig. 2A). To examine cell cycle distribution, we performed flow cytometry and found that RP11-89 depletion in 5637 Cell induced cell cycle arrest in S phase (Fig. 2B). Scratch and Transwell assays revealed that knockdown of RP11-89 markedly suppressed cell migration capacity compared with controls (Fig. 2C, D).

To further explore the effect of RP11-89 on BLCA, we conducted functional assays on T24 Cell with RP11-89 over-expression lentivirus as well as control. CCK8 assay showed that T24 Cell infected with lv-RP11-89 lentivirus exhibited enhanced cell viability compared with cells infected with lvNC-RP11-89 lentivirus (Fig. 2E). Flow cytometry showed that RP11-89 overexpression in T24 Cell inhibited cell cycle arrest in the S phase (Fig. 2F). Scratch and Transwell assays revealed that overexpression of RP11-89 markedly increased cell migration capacity compared with the control cells (Fig. 2G, H). We next constructed a subcutaneous xenograft model to examine the oncogenic function of RP11-89 in vivo (Fig. 2I). In tumors derived from cells transduced with sh-RP11-89 lentivirus, the tumor volume and weight were remarkably decreased and the rate of tumor growth was reduced compared with the control groups. Taken together, these results indicate that RP11-89 is an oncogenic lncRNA that promotes cell proliferation, migration capacity and inhibits cell cycle arrest in vitro and promotes BLCA tumorigenesis in vivo.

### RP11-89 induces iron export and ferroptosis resistance in BLCA

**Fig. 3:**
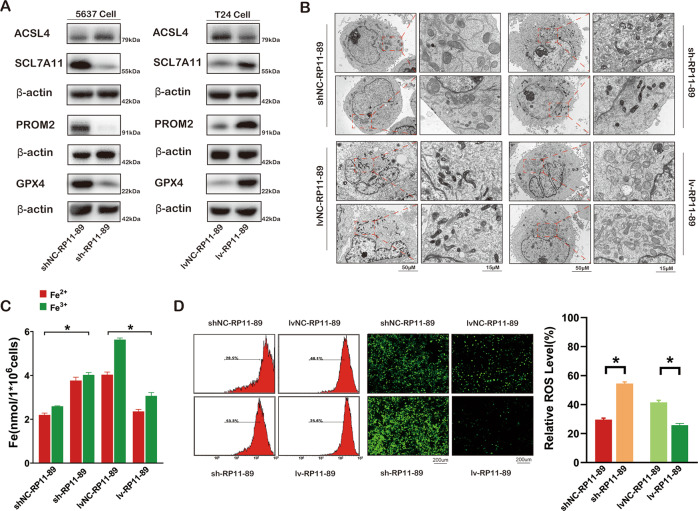
RP11-89 induces iron export and ferroptosis resistance in BLCA. **A** Western blot showed expressions of the ferroptosis-related proteins (ACSL4, SCL7A11, PROM2, and GPX4) in 5637 Cell with RP11-89 knockdown and T24 Cell with RP11-89 overexpression as well as control. **B** TEM analysis was performed to evaluate alterations in mitochondrial morphology in BLCA cells after RP11-89 knockdown or overexpression. **C** Iron content increased after RP11-89 knockdown and decreased after RP11-89 overexpression. **D** Confocal microscopy and flow cytometry of ROS levels in BLCA cells after RP11-89 knockdown or overexpression. Results are presented as mean ± SD. *P < 0.05, **P < 0.01, ***P < 0.001, ****P < 0.0001. Data were obtained from at least three independent experiments.

The high throughput mRNA sequencing results in Fig. 1 suggested that RP11-89 may regulate ferroptosis in BLCA. Furthermore, western blot analysis in Fig. 3A revealed that PROM2, SLC7A11, and GPX4 expressions were decreased in 5637 Cell infected with sh-RP11-89 lentivirus compared with control and increased in T24 Cell infected with lv-RP11-89 compared with control. The opposite trends were observed in ACSL4 expressions. TEM revealed that RP11-89 depletion in BLCA cells resulted in shrunken mitochondria and other ferroptotic features of mitochondria such as increased membrane density and markedly decreased mitochondrial cristae compared with control cells (Fig. 3B). Iron accumulation was increased in 5637 Cell infected with sh-RP11-89 lentivirus compared with control and decreased in T24 Cell infected with lv-RP11-89 compared with control (Fig. 3C). Analysis of ROS by confocal microscopy and flow cytometry showed a similar trend as the iron accumulation results (Fig. 3D). Together, these results suggested that RP11-89 plays a negative regulatory role in ferroptosis in BLCA.

### RP11-89 functions as an oncogene by sponging miR-129-5p

**Fig. 4:**
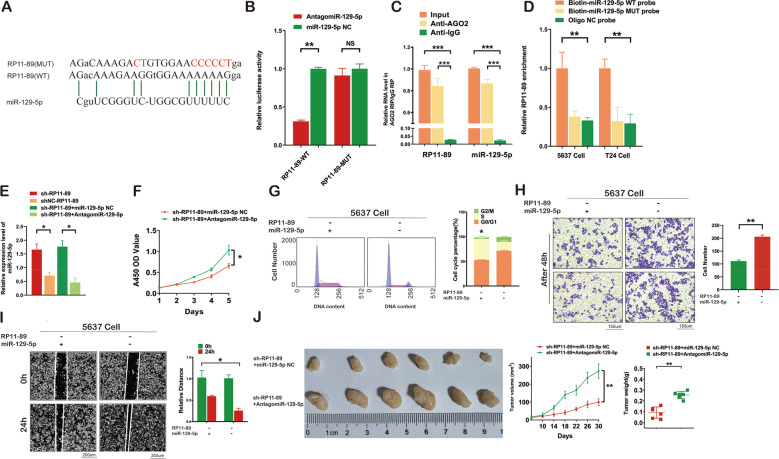
RP11-89 promotes tumorigenesis via sponging miR-129-5p in BLCA. **A** The binding sites between miR-129-5p and MUT/WT of RP11-89. **B** Dual luciferase assay showed that RP11-89 down-regulated miR-129-5p and miR-129-5p downregulated PROM2. **C** RIP assay confirmed the interaction of miR-129-5p with RP11-89 in BLCA cells. **D** RNA pull-down assay showed that RP11-89 was enriched in the sample pulled down by the Bio-miR-129-5p WT probe in 5637 Cell and T24 Cell. **E** The expression level of miR-129-5p in each group transfected as indicated was verified through RT-qPCR. **F** CCK-8 assay indicated that inhibition of miR-129-5p restored cell proliferation in RP11-89 knockdown cells. **G** Cell cycle analysis indicated the percentage of G2/M phase, S phase and G0/G1 phase cells in each group transfected as indicated. **H** Transwell assay revealed that inhibition of miR-129-5p restored the migration ability in RP11-89 knockdown cells. **I** Scratch test showed the migration capacities of cells transfected as indicated. **J** Representative images of tumors from nude mice inoculated with BLCA cells and inhibition of miR-129-5p increased tumor volume and tumor weight of nude mice after inoculation. Results are presented as mean ± SD. *P < 0.05; **P < 0.01, ***P < 0.001, ****P < 0.0001. Data were obtained from at least three independent experiments.

Potential downstream miRNAs of RP11-89 were identified using the miRcode database (Table 2), which indicated that RP11-89 might play a regulatory role via means of miRNAs. Among these miRNAs, specifically miR-129-5p has been previously reported to exhibit anti-tumor involvement in a variety of cancers [26, 27]. In addition, multiple critical regulatory genes of ferroptosis such as ACSL4 and PROM2, are targeted by miR-129-5p according to subsequent intersection analyses. Furthermore, ACSL4 and PROM2 are also dysregulated in high throughput sequencing results (Fig. 1G and Supplementary Table S1), which indicated the strong link between miR-129-5p and ferroptosis in BLCA. Therefore, we focus on miR-129-5p as a potential target of RP11-89 in current study. The potential binding sites between RP11-89 and miR-129-5p using bioinformatic methods was shown in Fig. 4A. Luciferase reporter assay was performed using luciferase reporter plasmids with WT and MUT of RP11-89. After transfection with agomiR-129-5p, the luciferase activities of WT-RP11-89 group were significantly reduced compared with cells transfected with miR-129-5p NC, while no significant differences were detected in MUT-RP11-89 group (Fig. 4B). We conducted immunoprecipitation assays using AGO2 antibody and found that the AGO2 antibody was able to enriched both endogenous miR-129-5p and RP11-89 (Fig. 4C). To examine the interaction between RP11-89 and miR-129-5p, we used biotin-labeled miR-129-5p WT/MUT probes to pull down endogenous RP11-89 in 5637 Cell and T24 Cell, respectively, followed by qRT-PCR. Compared with the oligo control probes, miR-129-5p WT probes enriched RP11-89 respectively while no significant enrichment was detected in miR-129-5p MUT probes (Fig. 4D). Furthermore, whether lncRNA RP11-89 was also co-located with miR-129-5p in subcellular level was critical for the validation of sponging interaction. As results of FISH assay and subcellular fractionation assay showed, with the nuclear control of U6 staining and the cytoplasm control of 18S staining, we observed that both RP11-89 and miR-129-5p are predominately located in cytoplasm in 5637 Cell and T24 Cell (Supplementary Fig. S1). Combined with convincing results of RIP assay, RNA pull-down assay and Dual luciferase assay, the validation of co-location provided further evidence for the sponging interaction between RP11-89 and miR-129-5p in subcellular level.

**Table 2:** Prediction of downstream miRNAs regulated by RP11-89 in miRcode database.

					Conservation		
microRNA family	Seed position	Seed type	Transcript region	Repeat	Primates (%)	Mammals (%)	Othervert. (%)
miR-137/137ab	chr2:45147710	7-mer-A1	ncRNA	No	33	0	0
miR-139-5p	chr2:45150352	7-mer-A1	ncRNA	No	22	0	0
miR-1ab/206/613	chr2:45149591	7-mer-A1	ncRNA	No	67	22	0
miR-200bc/429/548a	chr2:45149952	7-mer-m8	ncRNA	No	89	57	23
miR-216b/216b-5p	chr2:45148251	7-mer-m8	ncRNA	No	33	0	0
miR-23abc/23b-3p	chr2:45148970	7-mer-m8	ncRNA	Yes	67	9	0
miR-24/24ab/24-3p	chr2:45158601	7-mer-m8	ncRNA	No	67	22	0
miR-25/32/92abc/363/363-3p/367	chr2:45147711	7-mer-A1	ncRNA	No	33	0%	0
miR-27abc/27a-3p	chr2:45157174	7-mer-A1	ncRNA	No	89	78	31
miR-34ac/34bc-5p/449abc/449c-5p	chr2:45149170	8-mer	ncRNA	No	67	0	0
miR-125a-5p/125b-5p/351/670/4319	chr2:45148700	7-mer-A1	ncRNA	No	56	4	0
miR-10abc/10a-5p	chr2:45147863	7-mer-m8	ncRNA	No	67	0	0
miR-455-5p	chr2:45158611	7-mer-m8	ncRNA	No	89	4	0
miR-129-5p/129ab-5p	chr2:45157262	7-mer-m8	ncRNA	No	89	57	23

To investigate the effects of sponging miR-129-5p in vitro, we performed rescue experiments. RT-qPCR showed that knockdown of RP11-89 increased the level of miR-129-5p in 5637 Cell, and co-transfection of antagomiR-129-5p restored the expression of miR-129-5p (Fig. 4E). CCK8 assay showed that 5637 cell co-treated with sh-RP11-89 lentivirus and miR-129-5p NC exhibited attenuated cell viability compared with 5637 Cell co-treated with sh-RP11-89 lentivirus and antagomiR-129-5p (Fig. 4F). To examine cell cycle distribution, we performed flow cytometry and found that 5637 cell treated with sh-RP11-89 lentivirus and miR-129-5p NC showed cell cycle arrest in S phase compared with 5637 Cell treated with sh-RP11-89 lentivirus and antagomiR-129-5p (Fig. 4G). Transwell assay and scratch assay revealed that the cell migration capacity of cells treated with sh-RP11-89 lentivirus and miR-129-5p NC was markedly suppressed compared with 5637 Cell treated with sh-RP11-89 lentivirus and antagomiR-129-5p (Fig. 4H, I).

We next constructed a subcutaneous xenograft model to examine the function of sponging miR-129-5p in vivo (Fig. 4J). In tumors derived from cells co-transduced with sh-RP11-89 lentivirus and antagomiR-129-5p, the tumor volume and weight were remarkably decreased and the rate of tumor growth was reduced compared with the control groups. Inhibition of miR-129-5p expression partly recovered the tumorigenesis ability of BLCA cell with RP11-89 knockdown. Taken together, these results indicate that RP11-89 function as an oncogene in vitro and vivo by sponging miR-129-5p.

### PROM2 is targeted by miR-129-5p and upregulated in BLCA

**Fig. 5:**
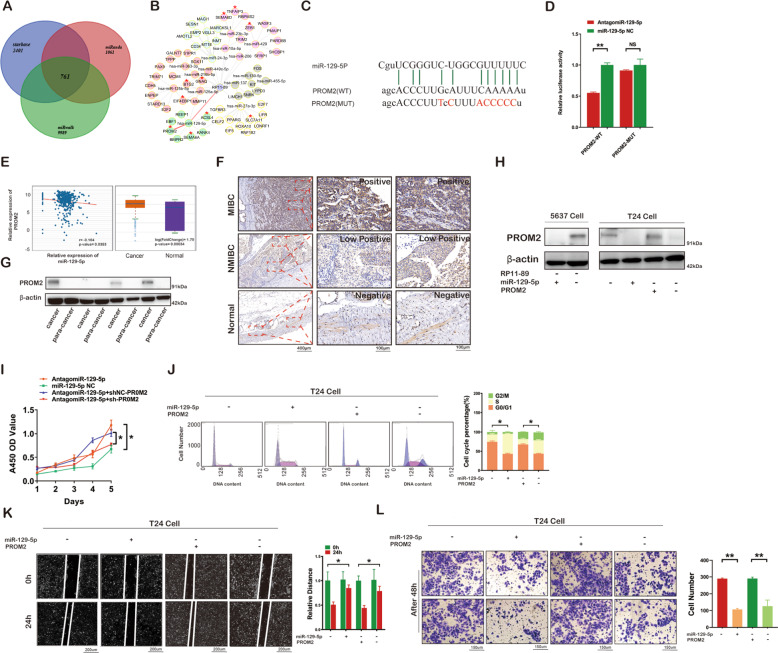
miR-129-5p suppresses bladder cancer via targeting PROM2. **A** Venn diagram shows target genes of miR-129-5p predicted by the starBase, miRanda and miRwalk databases. **B** The lncRNA-miRNA-mRNA regulatory networks show the association between RP11-89 and ferroptosis. *ferroptosis-related genes. **C** The binding sites between miR-129-5p and WT/MUT of 3′UTR of PROM2. **D** Dual luciferase assay showed that miR-129-5p down-regulated PROM2. **E** The upregulated expression of PROM2 in BLCA tissues and the negative correlation between miR-129-5p and PROM2 expression in TCGA database. **F** Immunohistochemistry analysis of PROM2 in normal tissues, NMIBC tissues and MIBC tissues assessed by Image J. **G** Western blot of the expression of PROM2 in four paired BLCA tissues. **H** Western blot confirmed the expression of PROM2 in each group transfected as indicated. **I** CCK-8 assay indicated that knockdown of PROM2 restored cell proliferation in miR-129-5p inhibited cells. **J** Cell cycle analysis indicated the percentage of G2/M phase, S phase and G0/G1 phase cells in each group transfected as indicated. **K** Scratch test showed the migration capacities of cells transfected as indicated. **L** Transwell assay revealed that knockdown of PROM2 restored the migration ability in miR-129-5p inhibited cells. Results are presented as mean ± SD. *P < 0.05; **P < 0.01, ***P < 0.001, ****P < 0.0001. Data were obtained from at least three independent experiments.

Using the starBase, miRanda and miRwalk databases, we obtained 761 target genes from intersection analyses (Fig. 5A) and combined with FerrDb database, we found that one of critical ferroptosis suppressors, PROM2 was a potential target of miR-129-5p. RP11-89 together with 15 miRNAs and 62 target mRNAs associated with ferroptosis including ACSL4, PROM2, SLC7A11, TNFAIP3, GNAQ, SEMA6D, SEMA6A, ZEB1, and EIF4EBP1, were used to construct the lncRNA-miRNA-mRNA regulatory network, which suggested strong links between RP11-89 and ferroptosis in BLCA (Fig. 5B). The potential binding sites between miR-129-5p and PROM2 3′-UTR in the starBase database was shown in Fig. 5C. Luciferase reporter assay was performed using luciferase reporter plasmids with WT and MUT of PROM2. After transfection with agomiR-129-5p, the luciferase activities of WT-PROM2 group were significantly reduced compared with cells transfected with miR-129-5p NC, while no significant differences were detected in MUT-PROM2 group (Fig. 5D). The expression level of PROM2 was elevated in BLCA tissues compared with non-tumor tissues and negatively correlated with the relative expression of miR-129-5p via analysis of the starBase database using TCGA cohort as reference (Fig. 5E). Furthermore, IHC analysis of PROM2 expression in MIBC, NMIBC, and normal bladder tissues (Fig. 5F) and Western blot analysis of PROM2 expression in BLCA and para-cancer tissues (Fig. 5G) from the FUSCC cohort showed consistent with TCGA results. These findings indicated that RP11-89 “sponges” miR-129-5p and miR-129-5p negatively regulates PROM2.

### miR-129-5p suppresses cell proliferation and migration capacity and induces cell cycle arrest via targeting PROM2 in BLCA

To investigate the effects of miR-129-5p/PROM2 axis in vitro, we performed rescue experiments. Western blot confirmed that inhibition of miR-129-5p increased the level of PROM2 in T24 Cell, and co-transfection of sh-PROM2 restored the expression of PROM2 (Fig. 5H). CCK8 assay showed that T24 Cell treated with miR-129-5p NC exhibited attenuated cell viability compared with T24 Cell treated with antagomiR-129-5p. The same trends were observed in T24 Cell co-treated with antagomiR-129-5p and shNC-PROM2 compared with T24 Cell co-treated with antagomiR-129-5p and sh-PROM2 (Fig. 5I). To examine cell cycle distribution, we performed flow cytometry and found that T24 Cell transfected with miR-129-5p NC showed cell cycle arrest in S phase compared with T24 Cell transfected with antagomiR-129-5p. The same trends were observed in T24 Cell co-transfected with antagomiR-129-5p and sh-PROM2 compared with T24 Cell co-transfected with antagomiR-129-5p and shNC-PROM2 (Fig. 5J). Transwell assay and scratch assay revealed that the cell migration capacity of T24 cells treated with miR-129-5p NC was markedly suppressed compared with T24 Cell treated with antagomiR-129-5p. The same trends were observed in the antagomiR-129-5p and shNC-PROM2 group compared with antagomiR-129-5p and sh-PROM2 group (Fig. 5K, L). The above results suggested miR-129-5p suppresses cell proliferation and migration capacity and induces cell cycle arrest via targeting PROM2 axis in BLCA.

### PROM2 promotes iron export and ferroptosis resistance via formation of multivesicular bodies (MVBs) in BLCA

**Fig. 6:**
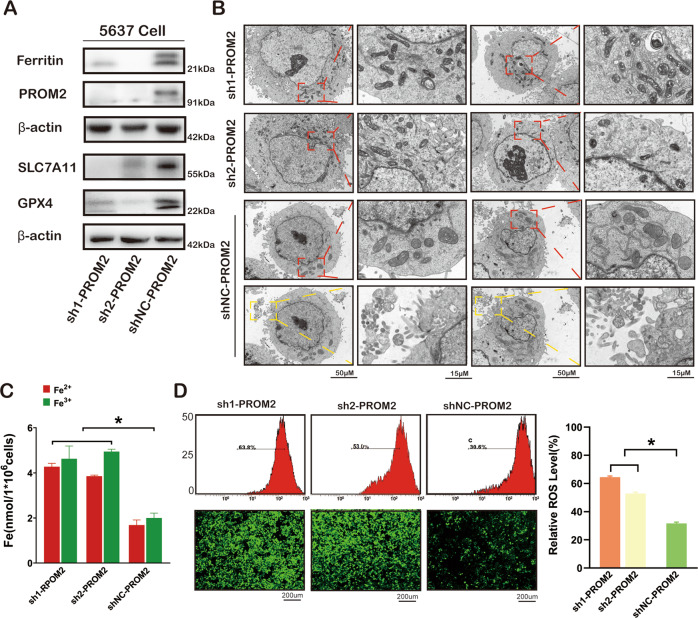
PROM2 induces iron export and inhibits ferroptosis via targeting the PROM2-Ferritin-MVBs pathway. **A** Western blot of the expression of GPX4, SCL7A11, Ferritin and PROM2 in 5637 Cell with PROM2 knockdown and the control. **B** TEM analysis showed shrunken mitochondria, increased membrane density and markedly decreased mitochondrial cristae (red boxes) in cells with PROM2 knockdown and formation of iron-containing multivesicular bodies with increased density was observed to induce iron export in control cells (yellow boxes). **C** Iron content increased after PROM2 knockdown. **D** Confocal microscopy and flow cytometry suggested that ROS levels increased after PROM2 knockdown. Results are presented as mean ± SD. *P < 0.05; **P < 0.01, ***P < 0.001, ****P < 0.0001. Data were obtained from at least three independent experiments.

Our results suggest that RP11-89 exerts an oncogenic effect in BLCA by acting as ceRNA of miR-129-5p and upregulating PROM2. As shown in Supplementary Fig. S2, the upregulation of ferroptosis related genes including PROM2, Ferritin, SLC7A11, and GPX4 was confirmed by immunohistochemistry analysis in BLCA tissues compared with normal tissues, which suggested that ferroptosis is associated with BLCA tumorigenesis. Furthermore, we next investigated the role of PROM2 in ferroptosis in BLCA. As shown in Fig. 6A, PROM2, Ferritin, SLC7A11, and GPX4 expressions were decreased in 5637 Cell infected with sh1-PROM2 or sh2-PROM2 lentivirus compared with 5637 Cell infected with shNC-PROM2 lentivirus. TEM revealed that PROM2 depletion in BLCA cells resulted in shrunken mitochondria and other ferroptotic features of mitochondria such as increased membrane density and significantly decreased mitochondrial cristae compared with control cells (Fig. 6B). Furthermore, 5637 Cell treated with control lentivirus contained more MVBs, an effect that was not observed in PROM2-silenced cells. Iron accumulation was decreased in 5637 Cell infected with sh1-PROM2 or sh2-PROM2 lentivirus compared with 5637 Cell infected with shNC-PROM2 lentivirus (Fig. 6C). Analysis of ROS by confocal microscopy and flow cytometry showed a similar trend as the iron accumulation results (Fig. 6D). Together these results suggested that RP11-89 facilitate ferroptosis resistance by formation of MVBs and iron export via regulating the PROM2-Ferrintin pathway (Supplementary Fig. S3).

## Discussion

Bladder cancer has recognized to be one of the most frequently mutated human cancers, following lung and skin cancer in mutation rates [28–30]. Recent studies have shown that lncRNAs regulate many important cancer phenotypes through controlling different cancer-related pathways [31, 32]. For example, Li et al. reported that lncRNA UBE2CP3 targets miR-138-5p to regulate gastric cancer progression via EMT process [33]. In this research, we demonstrated that lncRNA RP11-89 could play a regulatory role in bladder cancer ferroptosis and progression directly via a novel ceRNA network of RP11-89/miR-129-5p/PROM2 axis.

Rescue assay proved that the regulatory network is dysregulated in BLCA tumorigenesis. Recent studies have revealed that miR-129-5p plays an anti-tumor effect in a variety of cancers, such as kidney cancer [27, 34]. PROM2 plays an important role in the manipulation of ferritin-mediated iron export, which could modify ferroptosis sensitivity of cancer cells. In breast carcinoma cells, PROM2 has been found to contribute to iron transportation and inhibits ferroptosis through the formation of ferritin-containing MVBs and exosomes [35]. Our research observed the high expression of PROM2 in BLCA patient tissues, suggesting PROM2 may play an essential role in bladder oncogenesis. Previous studies suggested the potential for PROM2 as a tumorigenic biomarker for lung and ovarian cancers [36]. However, no research has investigated the role of PROM2 in BLCA and its association with miRNAs. In this study, we found that PROM2 expression negatively correlated with miR-129-5p expression and subsequently influenced BLCA tumorigenesis via regulating ferroptosis.

Increasing studies have investigated the role of ferroptosis, one of nonapoptotic forms of cell death in tumor cells [14]. Recent reviews by Chen [13] and Jiang [8] described the strong association of ferroptosis with tumorigenesis through various mechanisms such as cell hypoxia, epithelial-to-mesenchymal transition, immune response and multiple metabolic pathways. For example, hypoxia has been the focus of many recent studies and is mainly activated by HIFs [37]. Agents that inhibit hypoxia have been explored as a ferroptosis-related strategy for tumor growth inhibition in clinical trials such as PT2385 [38] in clear cell renal carcinoma. The increased production of ROS from hypoxia dysregulation directly leads to lipid peroxidation by disrupting the integrity of mitochondrial membranes during ferroptosis. In the current study, the high-throughput sequencing results indicated that RP11-89 functions as a ferroptosis-related oncogene through multiple cancer pathways including the HIF-1 signaling pathway, Hippo signaling pathway, adherens junction, cell cycle alteration, and multiple metabolic pathways.

In addition, recent studies have shown that ferroptosis strategy is sensitive to bladder cancer. For example, a novel agent called AuNRs&IONs@Gel constructed by Pengyu Guo was reported to induce ferroptosis and trigger a potent immune response through a triple therapy strategy using FDA-approved nanoparticles in BLCA [39]. The relationship between immune statue and ferroptosis in BLCA has been explored by accumulating studies [40, 41]. Luan et al. found that immune cell infiltration and immune checkpoints in BLCA were dysregulated in BLCA patients with different levels of ferroptosis related genes using multiple experimental and bioinformatic models [41]. The immune microenvironment is critical in BLCA treatments, especially in immunotherapies such as BCG instillation and immune checkpoint inhibitor [42, 43]. We previously identified a novel immune related lncRNA signature with prognostic significance for BLCA and RP11-89, one of lncRNAs in the signature, was demonstrated to play an oncogenic role through inhibiting regulated cell death [25]. Chen et al. demonstrated that the underlying mechanism for ferroptosis in immune regulation could be explained by immune surveillance, which indicated that tumor cell ferroptosis and tumor suppress could be triggered or enhanced by immune interventions [13]. In our research, we firstly demonstrated that the immune related lncRNA RP11-89 also functions as a negative regulator of ferroptosis during bladder tumorigenesis.

Although we have explored the role of RP11-89 in BLCA ferroptosis, the further mechanism leading to RP11-89 upregulation in BLCA remains to be fully illustrated. Whether lncRNA RP11-89 could play a regulatory role in BLCA immunity via interacting with PROM2 remains to be further investigated.

In conclusion, it is our novel discovery that RP11-89 induces tumor cell proliferation and migration, promotes tumorigenesis and inhibits cell cycle arrest via the miR-129-5p/PROM2 axis in BLCA. Furthermore, we demonstrated that RP11-89 function as a ceRNA against miR-129-5p and upregulates PROM2 expression, which contributes to ferroptosis resistance driven by a prominin2-MVB-exosome-ferritin pathway and iron export [35]. The current study indicates that RP11-89 is a promising molecule for bladder carcinogenesis and may contribute to the ferroptosis strategy for BLCA-targeted therapy in the future.

## Materials and methods

### Patients and samples

A total of 49 paired BLCA tissues and adjacent non-tumor bladder mucosal tissues were obtained from patients diagnosed with BLCA who underwent radical cystectomy at Fudan University Shanghai Cancer Center (FUSCC) from 2019 to 2021. This study was approved by the FUSCC Ethics Committee. All patients provided informed consent for participation in this study. The diagnosis of the enrolled patients was independently confirmed by at least two experienced pathologists at our center.

### Downstream microRNAs and target genes prediction

Downstream miRNAs regulated by RP11-89 were predicted using the miRcode database (https://cancergenome.nih.gov/) [44]. We obtained the predicted target genes of miR-129-5p from the starBase (http://starbase.sysu.edu.cn/panGeneDiffExp.php) [45], miRanda (https://omictools.com/miranda-tool) [46] and miRwalk (http://mirwalk.uni-hd.de/) [47] databases. We obtained the ferroptosis-related genes from the FerrDb database (http://www.zhounan.org/ferrdb/) [48]. A lncRNA-miRNA-mRNA regulatory network with ferroptosis-related genes was generated using Cytoscape 3.5.1 (https://cytoscape.org/) [49].

### Next-generation mRNA sequencing

We extracted total RNA from sh-RP11-89 5637, shNC-RP11-89 5637, lv-RP11-89 T24, and lvNC-RP11-89 T24 cell lines. The mRNA expression profile was obtained using the next generation sequencing conducted by Sangon Biotech (Shanghai, China). The differential expression of genes listed in the hierarchical clustering map was defined by the ratio of expression in lv-RP11-89 T24 Cell to that in lvNC-RP11-89 T24 Cell or the ratio of the expression in sh-RP11-89 5637 Cell to that in shNC-RP11-89 5637 Cell as a log2|fold change|≥ 1. The gene expression patterns in different pathways were analyzed using the KEGG pathway database (https://www.genome.jp/kegg/pathway.html) [50]. Subsequently, Western Blot and qRT-PCR were performed to validate the differential expression of relative genes (Supplementary Methods, Supplementary Table S2, and Supplementary Table S3).

### Nuclear-cytoplasm separation assay

Cells (at least 1 × 10^6^ cells) were collected and washed using PBS solution multiple times. We performed nuclear-cytoplasm separation assay using PARIS Kit (Life Technologies, USA) according to the manufacturer’s instructions. The expression levels of RP11-89, miR-129-5p as well as controls (GAPDH, β-Actin and U6) in nuclear and cytoplasm were detected by qRT-PCR.

### RNA immunoprecipitation (RIP) assay

The RIP kit (Millipore) was used to examine the RP11-89 and miR-129-5p interaction. Rabbit IgG (Millipore) served as the negative control. Immunoprecipitated RNAs from the total RNA (input control), IgG (NC) and Ago2 (ab186733, 1:50) groups were analyzed by qRT-PCR. The presence of RP11-89 and miR-129-5p was detected using specific designed primers. Sequences of RP11-89 and miR-129-5p are listed in Supplementary Table S4 and primer sequences are listed in Supplementary Table S2.

### Subcutaneous xenograft model

The male nude mice (BALB/c, aged 4–6 weeks, 18–20 g) were randomly divided into four groups (Sample size: 5–7 mice per group) and inoculated with cells as follows: sh-RP11-89 stable transfected 5637 Cell (1 × 10^7^ cells); shNC-RP11-89 stable transfected 5637 Cell (1 × 10^7^ cells); sh-RP11-89 stable transfected 5637 Cell + antagomiR-129-5p (1 × 10^7^ cells; 10 nmol antagomiR-129-5p injection/mouse, 3 days after tumor formation); and sh-RP11-89 stable transfected 5637 Cell + miR-129-5p NC group (1 × 10^7^ cells; 10 nmol miR-129-5p NC injection/mouse, 3 days after tumor formation). Cells were mixed with matrigel (1:2) and inoculated subcutaneously at the right rear back region. Tumor size was measured by calipers every 4 days and tumor volume was calculated as: volume = length × (width)^2^/2. Procedures involving animals were performed with approval from the Animal Care and Use Committee of the Medical Institution of Fudan University Shanghai Cancer Center.

### Statistical analysis

All experiments were carried out at least three times in triplicate. All the statistical tests were justified as appropriate. Analysis of variance was performed and assumption criteria were met and analysis of variance was performed. Results were expressed as mean ± standard deviation (SD). The experimental data were analyzed using statistical analysis software including GraphPad Prism 8.0 software (GraphPad) and the R package (V.3.3.4). Data are reported including estimation of variation within each group. Unpaired *t*-test or one-way ANOVA was used to measure differences between groups. Chi square (*χ*^2^) tests compared categorical variables. Statistical significance was determined at *P* < 0.05.

## Supplementary information

Figure S1

Figure S2

Figure S3

Table S1

Table S2

Table S3

Table S4

Supplementary materials

## Data Availability

The data and materials that support the findings of current study are available from the corresponding authors upon reasonable request.
