# Supplementary figures and images for: LncRNA RP11-89 facilitates tumorigenesis and ferroptosis resistance through PROM2-activated iron export by sponging miR-129-5p in bladder cancer
Source: Cell Death Dis. 2021 Nov 2;12(11):1043. doi: 10.1038/s41419-021-04296-1 (PMC8563982; doi:10.1038/s41419-021-04296-1)

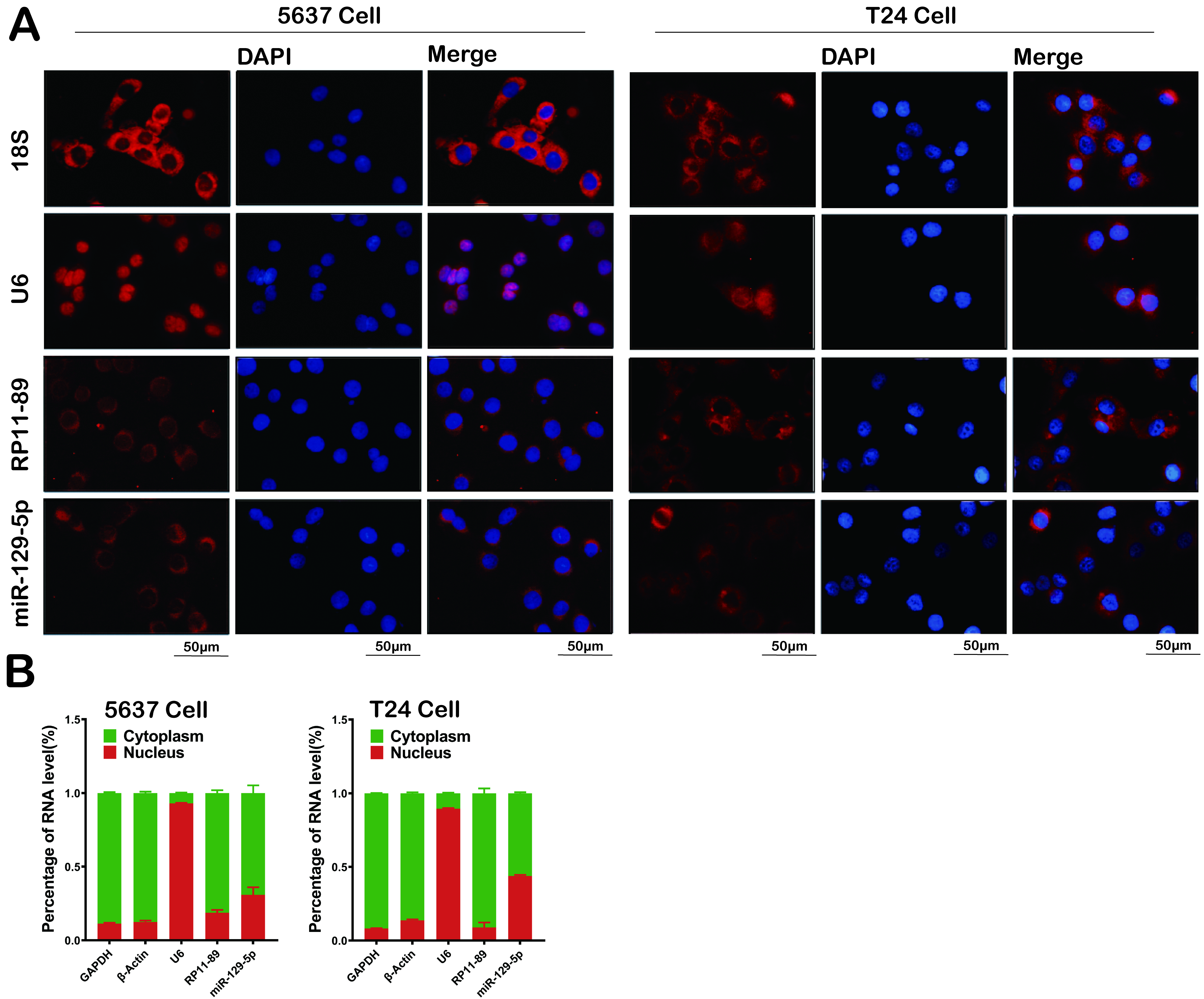

Supplement: Supplementary file 1 — Figure S1 [file 41419_2021_4296_MOESM1_ESM.tif]

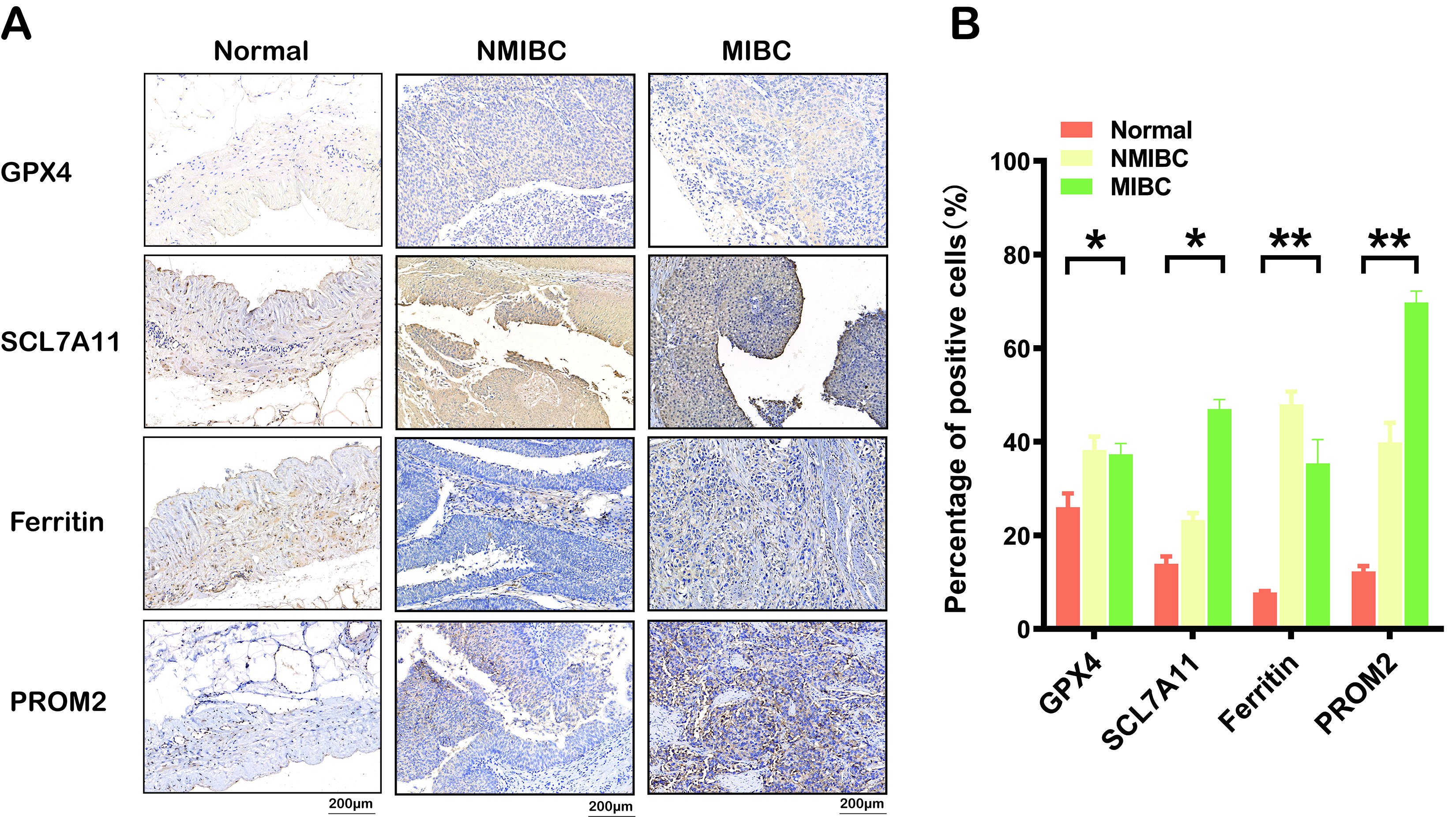

Supplement: Supplementary file 2 — Figure S2 [file 41419_2021_4296_MOESM2_ESM.tif]

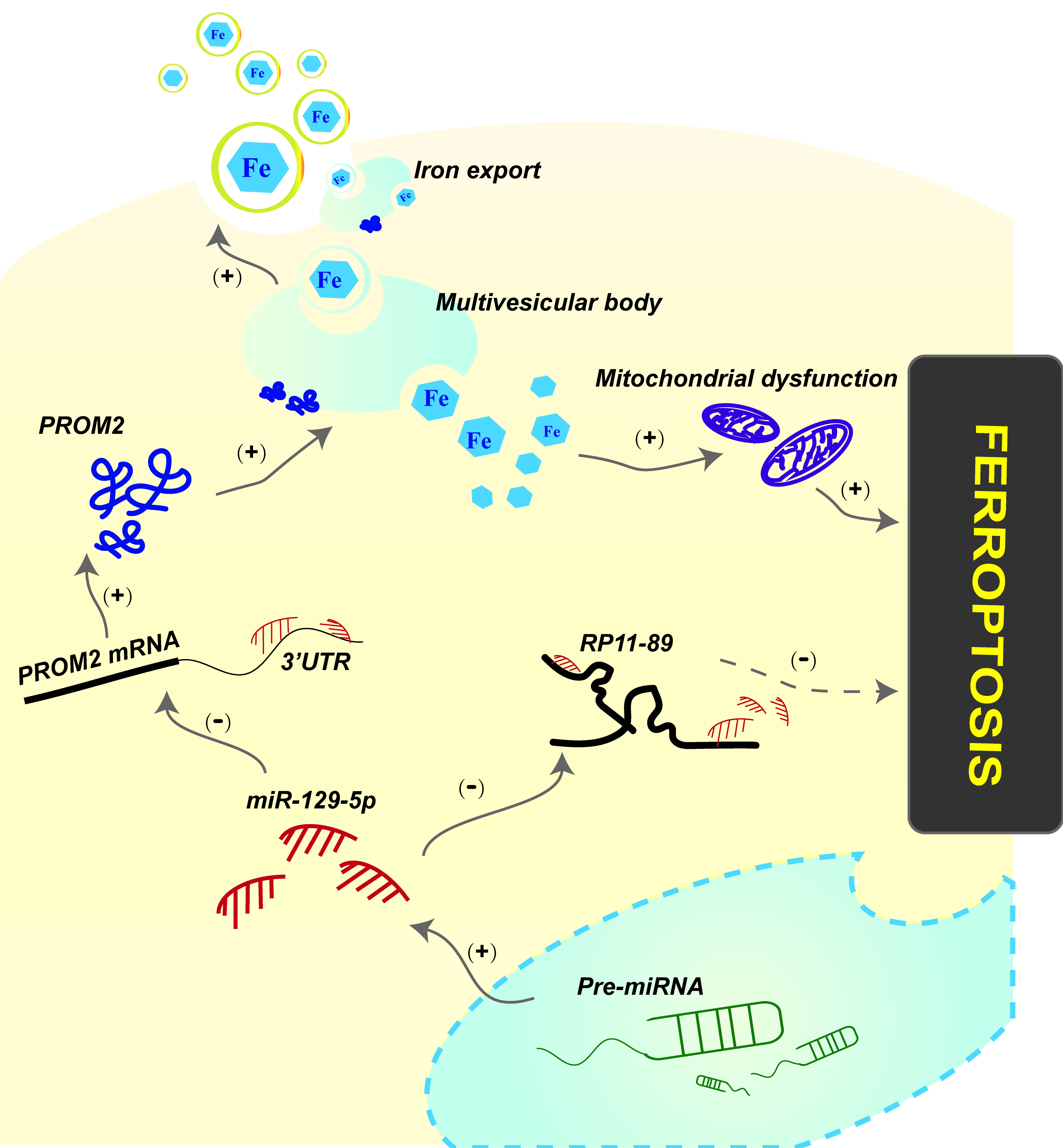

Supplement: Supplementary file 3 — Figure S3 [file 41419_2021_4296_MOESM3_ESM.tif]
