# Supplementary material for: LncRNA RP11-89 facilitates tumorigenesis and ferroptosis resistance through PROM2-activated iron export by sponging miR-129-5p in bladder cancer
Source: Cell Death Dis. 2021 Nov 2;12(11):1043. doi: 10.1038/s41419-021-04296-1 (PMC8563982; doi:10.1038/s41419-021-04296-1)
Supplement: Supplementary file 5 — Table S2 [file 41419_2021_4296_MOESM5_ESM.docx]

Table S2. The sequences (5'-3') of primers and shRNA target sequences related to methods.

| Primers | 5’ to 3’ |
| --- | --- |
| human miR-129-5p Forward primer | GGCTTTTTGCGGTCTGG |
| human miR-129-5p Reverse primer | CAGTGCGTGTCGTGGAGT |
| human PROM2 Forward primer | GGGCCACAGACTGCAAGTT |
| human PROM2 Reverse primer | AGCTCATTCAGTAGGGCCTTTA |
| human RP-11-89 Forward primer | TTGCGCAAGGTGGACACACAA |
| human RP-11-89 Reverse primer | GTCTGGCATGGTCAGAGAAAGAA |
| human GAPDH Forward primer | TGGGCGGCATGATTTCCTC |
| human GAPDH Reverse primer | GCCAGGACATTGTTGACCAG |
| sh1-RP-11-89 target sequence | GTGGCCGGCACATCTTCTGAG |
| sh2-RP-11-89 target sequence | GCCACAGGAGGACATGTTTCT |
| shNC-RP-11-89 target sequence | TTCTCCGAACGTGTCACGT |
| sh1-PROM2 target sequence | CCTCCAAATACTTCCGTCCTA |
| sh2-PROM2 target sequence | GCACCTGGATATCAACCAGTA |
| shNC-PROM2 target sequence | TTCTCCGAACGTGTCACGT |
|  |  |
