# Supplementary material for: LncRNA RP11-89 facilitates tumorigenesis and ferroptosis resistance through PROM2-activated iron export by sponging miR-129-5p in bladder cancer
Source: Cell Death Dis. 2021 Nov 2;12(11):1043. doi: 10.1038/s41419-021-04296-1 (PMC8563982; doi:10.1038/s41419-021-04296-1)
Supplement: Supplementary file 6 — Table S3 [file 41419_2021_4296_MOESM6_ESM.docx]

Table S3. Antibodies related to Western blot analysis and Immunohistochemistry

| Antibodies | Dilution and Source |
| --- | --- |
| ACSL4 | 1:1000 (WB), 1:250 (IHC); Abcam Cat# 155282 |
| SCL7A11 | 1:1000 (WB), 1:200 (IHC); Proteintech Cat# 26864-1-AP |
| PROM2 | 1:2000 (WB), 1:50 (IHC); Novus Biologicals Cat#NBP1-47938 |
| GPX4 | 1:2000 (WB), 1:2000 (IHC); Proteintech Cat# 67763-1-Ig |
| Ferritin | 1:1500 (WB), 1:600 (IHC); Abcam Cat# ab69090 |
| β-actin | 1:5000 (WB) Abcam Cat# ab6276 |
| Rabbit Anti-Rat IgG H&L (HRP) | 1:10000 (WB); Abcam Cat# ab6734 |
| Rabbit Anti-Mouse IgG H&L (HRP) | 1:10000 (WB); Abcam Cat# ab6728 |
|  |  |
