# Supplementary material for: LncRNA RP11-89 facilitates tumorigenesis and ferroptosis resistance through PROM2-activated iron export by sponging miR-129-5p in bladder cancer
Source: Cell Death Dis. 2021 Nov 2;12(11):1043. doi: 10.1038/s41419-021-04296-1 (PMC8563982; doi:10.1038/s41419-021-04296-1)
Supplement: Supplementary file 7 — Table S4 [file 41419_2021_4296_MOESM7_ESM.docx]

Table S4. The sequences of RP11-89, miR-129-5p, PROM2 3’UTR.

| Target | Sequence (5’ to 3’) |
| --- | --- |
| RP11-89 | GCGGTCCAGCCGCCGCCAGCGCCTTTCGGCTCTGGCCTGAAGCAGTACTCCCGCCTCCTGGAGCGGCCGCCCGATCCTAGCGCGGAGCTGCGAGCCTGACCGGCCGCGTCTGGCATGGTCAGAGAAAGAATTTTCTTTTCCCAACTCCGGCTTTTGGTTTTGTGTGTCCACCTTGCGCAACTCCGGAGCCAGCCGACCCCACATGGATTCTCAACAGGTGGCCGGCACATCTTCTGAGCCTCGCTCTCTCATCTGAAAGTGGAGTGTAAGTCCAAGAAGATTCATTTAGACAAAGAAGGTGGAAAAAAAGGACTTTCTGGGCCAGCAAGTCGGATGACCACCCTCCAAGGGGCAGAGGAGGGCCCATTTTGTGAAGAAGAAATCAACTACCCGGAAAACGCCACAGGAGGACATGTTTCTGCAGATGTAGTTGCCCTAGAAACAGAAGAGTATGGGGGTGTGAATGTCTTCTCTTTTGGGGGCAAACACTATGTCCTTTTCTTTTTCTAGATACAGTTAATTCCTGGAAATTTTAGCGAGTTTGTTCTTGTGGATATTTTGAACAATAAAGAGTGAAAATCAAAAAAAAAAAAAAAAAA  (NCBI Reference Sequence: NR_147194.1) |
| miR-129-5p | CUUUUUGCGGUCUGGGCUUGC |
| PROM2 3’UTR | GCAACAAGTTTTCTACTGGGAATTAGAATGGTGCATACACAATGTATTATTATCACTGTCAGATGAGCATGCTTGAATGTAGCATGACTGCCTCTTTTTGCTTTTCCTAGAGGTTTTTTTTTTGCTTGTTACTCATCTGTTGACCTACCTGGGGGAAGTAGCACCCTTGCATTTCAAAAATAAAATTGATGGCATTACAAATGGAA |
|  |  |
