# Supplementary material for: LncRNA RP11-89 facilitates tumorigenesis and ferroptosis resistance through PROM2-activated iron export by sponging miR-129-5p in bladder cancer
Source: Cell Death Dis. 2021 Nov 2;12(11):1043. doi: 10.1038/s41419-021-04296-1 (PMC8563982; doi:10.1038/s41419-021-04296-1)
Supplement: Supplementary file 8 — Supplementary materials [file 41419_2021_4296_MOESM8_ESM.docx]

**Supplementary Figure legends：**

Figure S1. The co-localization of RP11-89 and miR-129-5p in subcellular level was observed in BLCA cell lines.

1. FISH assay showed the subcellular localization of RP11-89 and miR-129-5p in 5637 Cell and T24 Cell with cytoplasm control (18S) and nucleus control (U6).
2. Subcellular fractionation assay validated that both RP11-89 and miR-129-5p were predominantly located in cytoplasm.

Figure S2. Immunohistochemistry analysis showed that ferroptosis related genes are upregulated in BLCA tissues.

A. Images of immunohistochemistry analysis of GPX4, SCL7A11, Ferritin and PROM2 in normal tissues, NMIBC tissues and MIBC tissues

B. The percentages of positive cells of GPX4, SCL7A11, Ferritin and PROM2 are measured using Image J.

Results are presented as mean ± SD. *P < 0.05; **P < 0.01, ***P < 0.001, ****P < 0.0001. Data were obtained from at least three independent experiments.

Figure S3. Diagram of mechanism and function of RP11-89. LncRNA RP11-89 acts as a sponge for miR-129-5p and upregulates PROM2 expression. PROM2 induces iron export via formation of PROM2-meditated Ferritin-containing multivesicular bodies, thereby inhibiting mitochondrial dysfunction and ferroptosis in BLCA cells.

**Supplementary Tables：**

Table S1. High throughput results of ferroptosis-related genes.

Table S2. The sequences (5'-3') of primers and shRNA target sequences related to methods.

Table S3. Antibodies related to Western blot analysis and Immunohistochemistry

Table S4. The sequences of RP11-89, miR-129-5p, PROM2 3’UTR.

**Supplementary methods：**

1. **Cells and cell culture**

Human BLCA cell lines T24 and 5637 were purchased from American Type Culture Collection. Cells were incubated in RPMI 1640 cell culture medium (Gibco) supplemented with 10% heat-inactivated fetal bovine serum and 50 μg/mL penicillin in a 37°C and 5% CO2 incubator. Cell lines were tested for mycoplasma contamination and showed no mycoplasma contamination.

1. **RNA isolation and quantification**

We isolated total RNA from cell lines and tissues from BLCA patients using the Trizol RNA isolation kit (Invitrogen). RNA concentration and purity were evaluated using a NanoDrop 2000c Spectrophotometer. Reverse-transcription reaction was conducted with the SuperScript First-Strand cDNA Synthesis System (Invitrogen). Primer sequences are listed in Supplementary Table S2. The ABI Prism 7900 Sequence Detector (Applied Biosystems) was used to perform real-time PCR with the SYBR Green PCR master mix in a 10 μL reaction mixture according to the manufacturer’s protocols. GAPDH mRNA served as the internal standard and we calculated the relative expression of genes by the 2-ΔΔCt method.

1. **Lentivirus preparation and infection**

Short hairpin RNA sequences (shRNA) against RP11-89 and PROM2 as well as scramble shRNA sequences were designed and cloned into the pLVX vectors following the manufacturer’s protocol. Lentivirus was produced in 293T cells by transient transfection with the above vectors. Lentiviral vectors for overexpression of RP11-89 and PROM2 were purchased from GeneChem. BLCA cell lines 5637 and T24 cells were infected with lentivirus and selected using 10 µM puromycin to establish stable transfected cell lines. The sequences of targets were listed in Supplementary Table S1 and Supplementary Table S2.

1. **Western blot analysis**

We extracted proteins from cells and tissues using RIPA lysis buffer (Beyotime Biotechnology). Protein samples were separated using 10% SDS-PAGE and transferred to 0.22 μm PVDF membranes (Millipore) under constant 220 mA in an ice bath for 2 h. The membrane was blocked with 5% skim milk powder and then incubated with primary antibodies at 4°C overnight. The membrane was then incubated with the appropriate secondary antibodies. After three 10 min washes with Tris-buffered saline containing 0.1% Tween 20 (TBST), the bands were detected using an ECL detection system (Bio-Rad), and β-actin was used as a loading control. The information of antibodies was listed in Supplementary Table S3.

1. **Cell transfection**

AgomiR-129-5p, antagomiR-129-5p and negative control (NC) were designed and purchased from GenePharma (Shanghai, China). Transfection was performed using Lipofectamine 3000 transfection reagent (Invitrogen).

1. **Scratch test and Transwell assay**

For scratch assays, cells were plated to 70% confluence on 6-well plates and incubated for 24 h. A straight line was scratched across the cell monolayer with 1-mL pipette tips, and plates were incubated for 24 h. The wound width was measured at 0 h and 24 h, and the plates were photographed.

For Transwell assays, cells were added to the upper chamber of a Transwell chamber filled with serum-free DMEM, and 600 μl of DMEM medium was added to the lower chamber. After incubation for 48 h, the cells were fixed with 4% paraformaldehyde and dyed with crystal violet (Sigma). Photos were taken under a digital microscope (Nikon).

1. **RNA pull-down assay**

Biotinylated miR-129-5p WT/MUT probes as well as the respective control (oligo probe) were pre-incubated with Streptavidin-Dyna beads M-280 (Invitrogen) at 25°C for 2 h. T24 Cell and 5637 Cell lysates were then incubated with the beads at 4°C overnight. After washing beads with wash buffer for multiple times, the RNA complexes bound to the beads were eluted and extracted using RNAiso Plus (TaKaRa) and RT- PCR was performed. The oligo probes were designed and synthesized by GenePharma (Shanghai, China).

1. **Transmission electron microscopy (TEM)**

Cells (at least 1 × 10^7^ cells) were collected and washed multiple times. After centrifugation, the cell pellet was mixed in agarose and fixed with 1% OsO_4_ (Ted Pella Inc.). Samples were dehydrated at room temperature multiple times using different concentrations of ethanol. After resin penetration and embedding, the samples were moved into a 65°C oven for polymerization, and the resin blocks were cut 60­–80 nm thin on an ultramicrotome (Leica UC7). Images of mitochondria were obtained using a HITACHI transmission electron microscope (HT7800/HT7700).

1. **Immunohistochemistry (IHC)**

IHC was conducted according to the manufacturer's instructions. The antibody and concentration are detailed in Supplementary Table S3. The staining result was viewed under an Olympus microscope (Tokyo, Japan) and analyzed using IHC profiles in ImageJ software (NIH).

1. **Cell viability and cell cycle analysis**

For cell viability assays, transfected or infected cells were seeded in 96-well plates (2,000 cells/well). After incubation for different time periods (1, 2, 3, 4 and 5 days), 10 μL of CCK8 solution (KeyGEN BioTECH) was added to each well, and cells were incubated for 2 h with 5% CO_2_ at 37°C. The absorbance of each well at 450 nm was measured using an automatic microplate reader (TECAN). Experiments were performed three times in duplicate.

Cell cycle analysis was performed using a cell cycle assay kit (KeyGen Biotech) according to the manufacturer’s instructions.

1. **ROS detection**

Intracellular ROS formation was measured using 2′7′-dichlorofluorescein diacetate (DCFH-DA). Results were observed with a confocal fluorescence microscope or flow cytometry analysis according to the manufacturer’s instructions (Thermo Fisher Scientific).

1. **Ferrous iron assay**

The intracellular ferrous iron level in cells was assessed with an iron colorimetric assay kit (Abcam) according to the manufacturer’s instructions.

1. **Dual-luciferase assay**

We designed and synthesized the wild-type (WT) and mutant (MUT) 3′-untranslated region (UTR) sequences of RP11-89 mRNA (WT-RP11-89, MUT-RP11-89) and PROM2 mRNA (WT-RPOM2, MUT-PROM2) and cloned the sequences into the pmirGLO dual-luciferase vector (Promega) to generate RP11-89 WT/MUT and PROM2 WT/MUT plasmids. Cells were then co-transfected with WT-RP11-89, MUT-RP11-89, WT-PROM2 or MUT-PROM2 together with miR-129-5p agomir. The luciferase activity of each group was evaluated using a Dual Luciferase Reporter Assay System (Promega) on a HT microplate reader (BioTek).

1. **Fluorescence in situ hybridization (FISH)**

Cells (at least 1 × 10^7^ cells in each sample) were prepared and fixed in 4% paraformaldehyde (DEPC). After digestion with proteinase K (Servicebio), samples were hybridized with RP11-89 and miR-129-5p probes (designed by Servicebio) and incubated overnight at 42°C. We removed the hybridization solution and washed sections in SSC (Servicebio). DAPI was used to stain cell nuclei, and images were taken using a fluorescence microscope (NIKON ECLIPSE CI).
